# Supplementary material for: MetaRibo-Seq measures translation in microbiomes
Source: Nat Commun. 2020 Jun 29;11:3268. doi: 10.1038/s41467-020-17081-z (PMC7324362; doi:10.1038/s41467-020-17081-z)
Supplement: Supplementary file 10 — Supplementary Data 7 [file 41467_2020_17081_MOESM10_ESM.zip › File2/Confidence_VeryHigh_Taxonomy/347617_out.krona.html]

Javascript must be enabled to view this page.

members
magnitude
magnitudeUnassigned
count
unassigned
taxon
rank

347617\_out

84

2
superkingdom
84

976
84
phylum

200643
84
class

171549
84
order

69
family
815

genus
69
3
816

SRS049995\_contig\_number\_32819SRS076976\_contig\_number\_contig-100\_8604.81635SRS893327\_contig\_number\_4001


SRS015431\_contig\_number\_15350SRS015486\_contig\_number\_399SRS015782\_contig\_number\_contig-100\_3222.237729SRS016132\_contig\_number\_393SRS016753\_contig\_number\_7296SRS017191\_contig\_number\_15986SRS017821\_contig\_number\_11923SRS017859\_contig\_number\_7853SRS018351\_contig\_number\_18521SRS020622\_contig\_number\_3296SRS020869\_contig\_number\_35367SRS022137\_contig\_number\_19126SRS023829\_contig\_number\_169SRS043667\_contig\_number\_contig-100\_1135.46700SRS043701\_contig\_number\_11343SRS046502\_contig\_number\_12935SRS046810\_contig\_number\_7SRS048164\_contig\_number\_31253SRS049773\_contig\_number\_30935SRS049959\_contig\_number\_contig-100\_955.269441SRS050752\_contig\_number\_19803SRS053649\_contig\_number\_8639SRS055982\_contig\_number\_11821SRS057480\_contig\_number\_3083SRS058268\_contig\_number\_4SRS062427\_contig\_number\_1494SRS063813\_contig\_number\_contig-100\_1079.58310SRS064557\_contig\_number\_4454SRS064973\_contig\_number\_12SRS075398\_contig\_number\_3239SRS075878\_contig\_number\_4645SRS077194\_contig\_number\_13118SRS077589\_contig\_number\_contig-100\_817.54225SRS077849\_contig\_number\_contig-100\_1217.191268SRS078176\_contig\_number\_23227SRS098073\_contig\_number\_14035SRS098881\_contig\_number\_26335SRS101376\_contig\_number\_4294SRS1041090\_contig\_number\_7961SRS1041133\_contig\_number\_29144SRS1054928\_contig\_number\_5665SRS1055049\_contig\_number\_16206SRS1055095\_contig\_number\_2207SRS142980\_contig\_number\_1811SRS143417\_contig\_number\_32293SRS143722\_contig\_number\_contig-100\_2373.86575SRS143780\_contig\_number\_7005SRS144537\_contig\_number\_51464SRS144603\_contig\_number\_6799SRS147557\_contig\_number\_contig-100\_796.196346SRS147614\_contig\_number\_1354SRS148319\_contig\_number\_8958SRS149325\_contig\_number\_12187SRS893172\_contig\_number\_9646SRS893187\_contig\_number\_430SRS893279\_contig\_number\_17007SRS893285\_contig\_number\_256SRS893366\_contig\_number\_27800SRS893369\_contig\_number\_7350SRS893380\_contig\_number\_2065SRS971275\_contig\_number\_11343
61
species
821

671267
1

SRS1041132\_contig\_number\_5984
species

species
2

SRS018817\_contig\_number\_250SRS075078\_contig\_number\_22200
818

626931

SRS143523\_contig\_number\_1305
1
species

820
species
1

SRS049446\_contig\_number\_6728

2005525
11
family

2
11
genus

SRS017745\_contig\_number\_contig-100\_2377.2378SRS148721\_contig\_number\_46288
375288

823
species
9

SRS015431\_contig\_number\_32441SRS019968\_contig\_number\_14529SRS045244\_contig\_number\_1577SRS046369\_contig\_number\_2031SRS048164\_contig\_number\_22655SRS051031\_contig\_number\_19501SRS056519\_contig\_number\_4231SRS063370\_contig\_number\_3865SRS077454\_contig\_number\_9929

171551
4
family

836
genus
4

658663
4
species

SRS015264\_contig\_number\_12000SRS019445\_contig\_number\_21542SRS043411\_contig\_number\_1603SRS147346\_contig\_number\_63731
